# Report of Three Cases of Aortic Aneurism*Read by invitation before the Pueblo County Medical Society, March 7, 1899.

**Published:** 1899-04

**Authors:** J. N. Hall

**Affiliations:** Denver, Colorado; Professor of Medicine in Gross Medical College; Visiting Physician to the Arapahoe County Hospital and St. Anthony’s Hospital


For Texas Medical Journal.

### Report of three Cases of Aortic Aneurism.*

BY J. N. HALL, M. D., DENVER, COLORADO.

Professor of Medicine in Gross Medical College; Visiting Physician to the Arapahoe County Hospital and St. Anthony's Hospital.

During the last year three cases of aortic aneurism came under my care affecting different portions of the aortic arch, and, taken together, they illustrate so many of the points of interest in this disease that I report them to you tonight.

CASE 1.—Joseph R., Austrian, miner, 45 years of age, was admitted to St. Anthony's Hospital March 31, 1898. His condition was so serious that I was asked to see him at once. At noon I found him suffering extremely from dyspnœa, so that he could not give a history of his illness. He was hoarse, and slightly cyanotic, pupils normal, pulse 100 to 112 but of fair quality, temperature 100°.

His complaint of suffering in his chest, with moderate cough, led to an immediate examination, with the following result:

The entire left chest was flat in front, the dullness passing from one to two inches beyond the median line. Heart beat not felt, but the sounds were most distinct in the fourth space to the right of the sternum. Over the normal cardiac region there was systolic friction. Breath sounds and vocal resonance were absent over the regions of flatness. In the left back there were resonance and fair respiratory sounds below, but flatness from the seventh rib upward with the other signs of pleural effusion already mentioned. I called the attention of Dr. Le Rossignol, the house physician, to the region of resonance at the base, indicating an adherent lung, stating that we should have to tap the patient, on this account, in the fifth space between the left nipple and the anterior axillary line as soon as I could return with the aspirator, since the one belonging to the hospital was out of order.

My diagnosis at this time was a left pleural effusion limited by old adhesions of the lower lobe to the posterior chest wall. I looked upon the pericardial friction as entirely secondary, and overlooked the possibility of aneurism entirely.

Prescribing a cathartic and cardiac stimulants, I left, expecting to return shortly to tap the chest. Dr. Le Rossignol telephoned me, soon after I reached my office, that the patient had just suffered a

*Read by invitation before the Pueblo County Medical Society, March 7, 1899.

frightful attack of dyspnœa, and I instructed him to tap the patient at once at the point which I had indicated to him without awaiting my arrival, even though the aspirator did not work well. I was at this time three miles from the hospital.

The withdrawal of nearly a pint of serous fluid gave immediate relief, the aspirator failing to work at this point. Before I arrived at the hospital, and some three hours after the tapping, the patient succumbed to a second attack of dyspnœa.

The autopsy was held twenty-two hours after death, by Dr. George H. Stover and myself. Two quarts of slightly blood-stained serum were found in the left pleural cavity. Left lung adherent behind up to the seventh rib, and fairly crepitant. The remainder of the lung was black, solid and airless. Right lung black, the authracosis being such as is commonly seen in miners, but the lung tissue approximately normal in gross appearance. The right lung had been crowded somewhat to the right by the effusion, the displaced heart, and the aneurism to be mentioned.

The heart was found in the center of the body, displaced by the pleural effusion. A dry plastic pericarditis was present. The organ was slightly enlarged, but in fairly normal condition in other respects.

Beginning one inch above the heart, but developing in such a direction as not to come in contact with the sternum, was an aneurism of the ascending and transverse portions of the aortic arch, of the size of a base-ball, nearly filled with thrombotic deposit. It was adherent everywhere, and encroached more upon the left pleura than the right. The liver, spleen, kidneys and other organs were normal in gross appearance. There were no visceral signs of syphilis. As I saw this patient but once during life, and as, owing to the great difficulty in speaking, he gave me practically no history, I am not surprised that I overlooked his original trouble. In fact, I doubt if it could have been differentiated from the pleural effusion and the pericarditis, unless pulsation, which I did not find, or tracheal tugging, which I did not look for, were present. I have little doubt, however, that before the advent of the pleural and pericardial troubles, from a tumor of the size mentioned, there must have been pressure signs referrable to the esophagus, trachea, vagus or sympathetic nerves, or other contiguous structures, which would have led to a correct diagnosis. Although it could have made no difference in the eventual outcome in this case, I have fairly made up my mind that I will never leave another case of large pleural effusion without tapping it, if I am permitted to do so, since one cannot tell when to expect an access of dyspnœa which may be fatal.

I regard the pleurisy in this case as secondary to the inflammatory processes around the advancing aneurism, and the pericarditis as having originated by extension from the left pleura.

For the history of the next case I shall quote from a report of a clinical lecture given upon it October 15, 1898, and published in the *Medical Fortnightly:*

Case 2.—Frank C., is a man of 50 years, a puddler in an iron foundry, without family history of interest in this connection. He was admitted September 25, 1898. He has used some liquor, and, more important in relation to his present illness, contracted syphilis eighteen years ago. His work has been laborious, involving much severe muscular effort. He was well until about a year ago, when, while at work for a time with a threshing machine, he was suddenly attacked with hoarseness, which developed into complete aphonia in a few days. He was admitted into a hospital in the State of Washington, remaining twenty-two weeks without material benefit. Being abvised to seek this climate, for supposed lung trouble, he came to Denver. Attacked upon the street with severe dyspnœa, he was brought to the hospital with the diagnosis, natural enough after a casual examination in the ambulance, of bronchial asthma.

When I saw him on September 25th his dyspnœa was so severe that no complete examination of the chest was made. Indeed, it seemed at first to be more properly a case for the laryngologist. The wheezing set up by the obstruction to the entrance of air was transmitted over the whole chest. He was relieved by morphia administered hypodermatically. The pupils were at this time equal. Several patches of syphilitic rupia existed at this time.

Dr. Levy reported that the left vocal cord was completely relaxed, and that the dyspnœa was therefore not of laryngeal origin. As no obstruction existed above the larynx the trouble was obviously below. Meanwhile the patient had materially improved under the use of iodide of potassium and bichloride of mercury, and occasional doses of morphia. On September 29th, the left pupil was moderately dilated. Pulse and temperature normal, the radial pulses equal. The respiratory murmur was moderately diminished over the whole left side, with diminution in the fremitus set up by the tracheal obstruction as compared with that of the right side. Heart negative. Urine negative. Marked tracheal tugging. Two days later the patient raised considerable blood in his expectoration, and has continued to do so at intervals since that time. Occasionally he is able to speak in a hoarse voice. He complains of some pain at times in the upper part of the chest. He has lost twenty-four pounds of flesh.

The diagnosis of aneurism in this case depends upon the fact that we have pressure symptoms originating from the region of the middle or transverse portion of the arch of the aorta. The paralysis of the left vocal cord depends upon pressure upon the left recurrent laryngeal nerve as it winds under the aortic arch. The dilatation of the left pupil, which is pretty constantly present in this case, depends upon pressure upon the last cervical or first dorsal sympathetic ganglion. The pressure, however, has thus far produced irritation, not complete paralysis, for in that case we should see contraction of the pupil with flushing and possibly sweating of the left side of the face. The dyspnœa, the tracheal tugging, the diminution in respiratory murmur, and non-transmission of the tracheal rale upon the left side, are accounted for by pressure upon the left primary bronchus. The expectoration of bloody mucus signifies that, from pressure, the mucous membrane of the affected bronchus has become so congested as to cause moderate hemorrhage.

No tumor is to be found, because the point which must be occupied by a tumor of any kind producing the results we have been studying is too far removed from the surface of the chest to permit of its detection by external examination, until it increases to a great size. The pain is accounted for by pressure of the enlarging aneurism upon neighboring nerves, and will probably increase if nothing occurs to prevent the continued growth of the tumor. It is probable that the aneurism does not involve the origins of any of the great branches springing from the arch, since no evidence is present of disturbance of the pulse in the arms, the neck or elsewhere. The absence of venous congestion and edema, and of clubbing of the nails upon either hand is sufficient to prove the absence of serious pressure upon any of the venous trunks. The patient is fortunate in being able to swallow without difficulty, for in many of these cases dysphagia from pressure upon the esophagus is a serious symptom.

The diagnosis of aneurism must be only a probable one for the present, since all the symptoms thus far noted excepting the tracheal tugging might arise from a tumor of the mediastinum exerting pressure upon the affected parts. The absence of enlargement of the cervical and clavicular glands, of notable cachexia, and of serious emaciation, and the fact that the patient has certainly had the present trouble for at least a year, all point toward aneurism, since most tumors of the mediastinum are of a malignant nature, the patient rarely surviving for a year after the first development of symptoms.

The prognosis is bad, as medicine offers little encouragement in such cases, and surgical intervention is out of question. I know of nothing better for the patient than the iodide of potassium, and the hypodermic use of morphia as required for the severe attacks of dyspnœa and pain.

On October 30th, the respiratory murmur upon the left side was fainter, but no rales were present. The pain was less in the chest, but was severe at times in the right shoulder.

On November 12th, the left radial pulse was weaker than the right. Dyspnœa and choking sensation worse. More cough. On November 24th, the heart was noted as having been gradually displaced to the left during the past few weeks, until it now beats two inches to the left of the left nipple in the fifth space. The left hand is congested and slightly cyanotic. Slight dullness exists over inner end of the left clavicle, and the cardiac second sound is easily heard at this point. No impulse can be detected.

On December 4th, slight dullness was found in the left back between the upper part of the scapula and the spine. No impulse.

On December 30th, for the first time, the chest could be felt to heave when pressed between the two hands antero-posteriorly. The right hand is considerably lighter in color than the left, and rather scaly. No change in the nails of either hand. No edema.

A recent examination of the patient has shown no particular change in his condition. His excellent flesh, color and general appearance after seventeen months of illness are scarcely consistent with the presence of a malignant growth in the chest, while the improvement seen in certain directions is not greater than we sometimes find in these cases under the influence of rest, good food and the only drug which is of much value, iodide of potassium. I believe that the aneurism now involves the descending portion of the arch, since it is evidently approaching the surface, at the upper part of the left back. Examination on March 3rd, by the fluorescope showed a shaded area about four inches across above the heart upon the left side, but the radiograph was not satisfactory, perhaps from insufficient exposure.

Case. 3.—S., colored male, 52 years of age, was admitted to my service at the Arapahoe County Hospital November 2, 1898. He was formerly a valet, but of late years has done some manual labor. He had syphilis when a youth, he states, although the history was not entirely clear. For three years he has complained of pain in the left side at times, and has been treated by several physicians for dry pleurisy. During much of this time he has been able to work.

He has had but little cough, with slimy expectoration. Although he has lost twenty-five pounds in weight, he is still in fair flesh. He came to Colorado from Missouri, for his health, last summer, and gained a little in flesh and strength for a short time. His chief complaint is of burning pain in the left lower back, upon inspiration, running forward along the ribs from the left scapular region, and of pronounced debility. The complaint of burning pain led me to examine with especial interest the upper portion of the left back, for such is certainly not the character of the pain of pleurisy. The scars of several blisters were to be seen in this region and over the left side. Between the left scapula and the spine was a point of the size of the palm of excessive tenderness, flat upon percussion. By side illumination it could be seen to project slightly, and to pulsate with the apex beat. The pulsation could also be felt, although not well marked. The cardiac sounds were audible here, and the second sound was very clear. There was great tenderness over the left ribs anterior to the outer border of the scapula.

The lower left chest was resonant, but the respiratory murmur was feeble. The heart was displaced toward the right, beating feebly at the left of the sternum in the fifth space. Sounds all feeble excepting the aortic second, which was very sharply accentuated. The right lung, the liver, and the other abdominal organs, were negative upon examination, as was also the urine. Tracheal tugging was present, but not as well marked as in many cases. Although the voice was squeaky in character, having formerly been full, laryngoscopic examination showed no paralysis of the vocal cords. The left pupil was larger than the right, and showed some old adhesions which distorted it. The radial and femoral pulses were equal, upon the two sides, and of good strength. The femoral pulse was not perceptibly delayed.

The diagnosis of aneurism of the descending portion of the arch and the adjoining thoracic aorta was made, and the patient ordered morphine for pain, and ten grain doses of iodide of potassium. A few days later he complained of dysphagia for the first time, and had a chill, with rise of temperature to 101°. His pulse thereafter ran from 92 to 132, and respirations from 24 to 32 per minute. Slight chills, without serious rise of temperature, occurred several times during the remainder of his life. On November 11th, the case was seen with me by Dr. H. W. McLauthlin. The tumor had increased greatly in size. Diastolic shock was noted for the first time. The dorsal spines were not tender upon percussion.

On November 13th, the left radial pulse was weaker than the right. Patient spoke only in whispers. The whole upper chest could be felt to heave with the cardiac pulsations upon making pressure between the two hands from before backward.

November 18th, no respiratory sounds in lower left lung. Resonance normal. On November 19th, two sharp hemorrhages following cough, and obviously coming from trachea. A pint of blood was lost in the two attacks. Tumor larger. Patient rapidly failing. November 20th, the patient suffered a third hemorrhage, and, upon entering the ward, was seen to be bleeding for the fourth time. He expired almost instantly. The blood, nearly a quart in amount, was slightly mixed with air.

The autopsy was performed twenty hours after death, by Dr. Ahlquist. The tumor in the left scapula region, noted during life, had disappeared. The heart was negative, possibly slightly smaller than usual. It was displaced about two inches to the right. Aorta atheromatous. At the beginning of the descending thoracic aorta was an egg-shaped aneurism three by four inches, which had eroded the sixth rib entirely through, and the fifth and seventh ribs partially. The adjacent vertebræ were slightly eroded. The aneurism had ruptured into the left primary bronchus and left lung, and a clot weighing a pound was found in the left pleural cavity near the heart. The upper portion of the cavity was obliterated by adhesions. The trachea and bronchi were filled with clotted blood. A well marked perisplenitis existed.

All other organs were normal in gross appearance.

It is interesting to notice that the heart was displaced toward the right in one of these cases, in part, at least, by the effusion; toward the right by the aneurismal tumor in one, and toward the left in one. It is to be expected that aneurisms of the ascending portion of the arch shall press the heart to the left, but the direction of the growth of the tumor in affections of other portions determines the direction of cardiac displacement.

In a case which I saw with Dr. H. W. McLauthlin last March, in which the aneurism projected through the chest wall above the left nipple, the heart was displaced to the right.

In conclusion, and especially inasmuch as I overlooked the condition in one of these cases in the single examination which I made, I know you will pardon the suggestion that we fail in the diagnosis of aortic aneurism, as in that of many other diseases, rather because we do not look, than because we do not know. In other words, we often fail to find plain signs of disease because we neglect to look for them, and thus fail in diagnosis.

